# The Tyrosine Phosphatase hPTPRβ Controls the Early Signals and Dopaminergic Cells Viability via the P2X_7_ Receptor

**DOI:** 10.3390/ijms222312936

**Published:** 2021-11-29

**Authors:** Francisco Llavero Bernal, Miriam Luque Montoro, Alazne Arrazola Sastre, Hadriano M. Lacerda, José Luis Zugaza

**Affiliations:** 1 Achucarro Basque Center for Neuroscience, Science Park of the UPV/EHU, 48940 Leioa, Spain; miriamluquem@gmail.com (M.L.M.); alazne.arrazola@ehu.eus (A.A.S.); 2 Research Institute of the Hospital 12 de Octubre (i + 12), 28041 Madrid, Spain; 3 Department of Genetics, Physical Anthropology, and Animal Physiology, Faculty of Science and Technology, UPV/EHU, 48940 Leioa, Spain; 4 Three R Labs, Science Park of the UPV/EHU, 48940 Leioa, Spain; hadrilac@gmail.com; 5 IKERBASQUE, Basque Foundation for Science, Plaza Euskadi, 48009 Bilbao, Spain

**Keywords:** P2X_7_ receptor, Ras GTPase, Erk1/2, hPTPRβ, dopaminergic neurons, Parkinson’s

## Abstract

ATP, one of the signaling molecules most commonly secreted in the nervous system and capable of stimulating multiple pathways, binds to the ionotropic purinergic receptors, in particular, the P2X_7_ receptor (P2X_7_R) and stimulates neuronal cell death. Given this effect of purinergic receptors on the viability of dopaminergic neurons model cells and that Ras GTPases control Erk1/2-regulated mitogen-activated cell proliferation and survival, we have investigated the role of the small GTPases of the Ras superfamily, together with their regulatory and effector molecules as the potential molecular intermediates in the P2X_7_R-regulated cell death of SN4741 dopaminergic neurons model cells. Here, we demonstrate that the neuronal response to purinergic stimulation involves the Calmodulin/RasGRF1 activation of the small GTPase Ras and Erk1/2. We also demonstrate that tyrosine phosphatase PTPRβ and other tyrosine phosphatases regulate the small GTPase activation pathway and neuronal viability. Our work expands the knowledge on the intracellular responses of dopaminergic cells by identifying new participating molecules and signaling pathways. In this sense, the study of the molecular circuitry of these neurons is key to understanding the functional effects of ATP, as well as considering the importance of these cells in Parkinson’s Disease.

## 1. Introduction

Purines may initiate signal transduction pathways in different cell lineages and tissues. These signaling networks are controlled by specific cell membrane receptors known as purinergic or purinoceptors [1]. They are classified into two types, metabotropic or P1 receptors and ionotropic or P2 receptors. Whereas P1 receptors bind heterotrimeric G proteins, ionotropic or P2 receptors can either bind heterotrimeric G proteins (P2Y) or form ion channels (P2X), exclusively activated by ATP, with selective permeability to Ca^2+^, Na^+^, and K^+^ [2]. The P2X receptors are expressed primarily in excitable cells, and seven subtypes (P2X_1–7_) [3] have been cloned. Amongst them, P2X_7_ is a purinergic ATP-binding receptor expressed at high levels in the central nervous system (CNS) [4,5,6,7]; therefore, it attracts the most attention [8].

The P2X receptor (P2X_7_R) is considered key in promoting an inflammatory response in pathological processes, such as neuroinflammation and neurodegeneration [6,9,10,11]. In this regard, there is evidence that the P2X_7_R pathway causes neuronal damage, leading to progressive neurodegeneration [11]. In fact, it has been reported that P2X_7_R antagonists can attenuate neuronal dysfunction and damage in a mouse model of Parkinson’s disease [12]. Additionally, selective inhibition of P2X_7_R also markedly protected dopaminergic (DA) neurons of the substantia nigra (SN) in rats [13,14,15].

ATP activation of the P2X_7_R stimulates multiple signaling pathways via its interaction with a complex of eleven different proteins, such as laminin α3, integrin β2, the receptor tyrosine phosphatase β (PTPRβ), α-actinin 4, β-actin, supervilin, heat shock proteins Hsp90, Hsc71, and Hsp70, phosphatidylinositol kinase 4 (PI4K), and membrane-associated protein guanylate kinase P55 (MaGuK) [16]. Two of the eleven proteins, PTPRβ and Hsp90, were proposed to modulate P2X_7_ receptor function [16,17]. These interactions allow the P2X_7_R to act as a transducer between the extracellular medium and the intracellular signaling pathways to modulate ion fluxes (Ca^2+^ and Na^+^ influx, K^+^ efflux), NADPH oxidase complex and reactive oxygen species (ROS) generation, and activation of phospholipase D, MAPKs (Erk1/2), p38, JNKs, and several caspases [18].

Ras GTPases control Erk1/2 serine/threonine kinases that regulate mitogen-activated cell proliferation connecting cellular responses to extracellular signals and playing an essential role on the modulation of a wide range of cellular processes both in health and diseases, such as cancer, and cardiopathies, including the neuronal responses [19] in neurocognitive and neurodegenerative diseases, such as Parkinson’s Disease [20,21,22,23]. The Ras GTPases function as molecular switches that cycle between an inactive GDP-bound and an active GTP-bound state. The transition between the inactive to the active state is regulated by three main molecules: guanine nucleotide exchange factors (GEF), GTPase activating proteins (GAP), and GDP dissociation inhibitors (GDI) [24].

Here, using SN4741 cells, a neuronal cell line derived from murine Substance Nigra [25], we showed that P2X_7_ receptor stimulation leads to Ras and Erk1/2 activation. By combining pharmacological and genetic approaches, we identified the tandem Ca^2+^-Calmodulin/Ras GRF1 activation as a bridge between the P2X_7_ receptor and the Ras/Erk1/2 pathway. We also show that PTPRβ tyrosine phosphatase controls not only the P2X_7_ receptor-mediated Ras activation, but it also controls dopaminergic cells viability. Overall, these results reveal unexpected crosstalk between the P2X_7_ receptor and the small GTPase Ras to modulate cell viability through PTPRβ. Interestingly, we demonstrated that dopaminergic neurons control of PTPRβ and Ras can maintain their viability. This being a central issue in palliating Parkinson’s Disease neurodegeneration.

## 2. Results

### 2.1. 0.5 mM ATP Mediates Erk1/2 Phosphorylation via the P2X_7_ Receptor without Affecting Cell Viability

**Figure 1:**
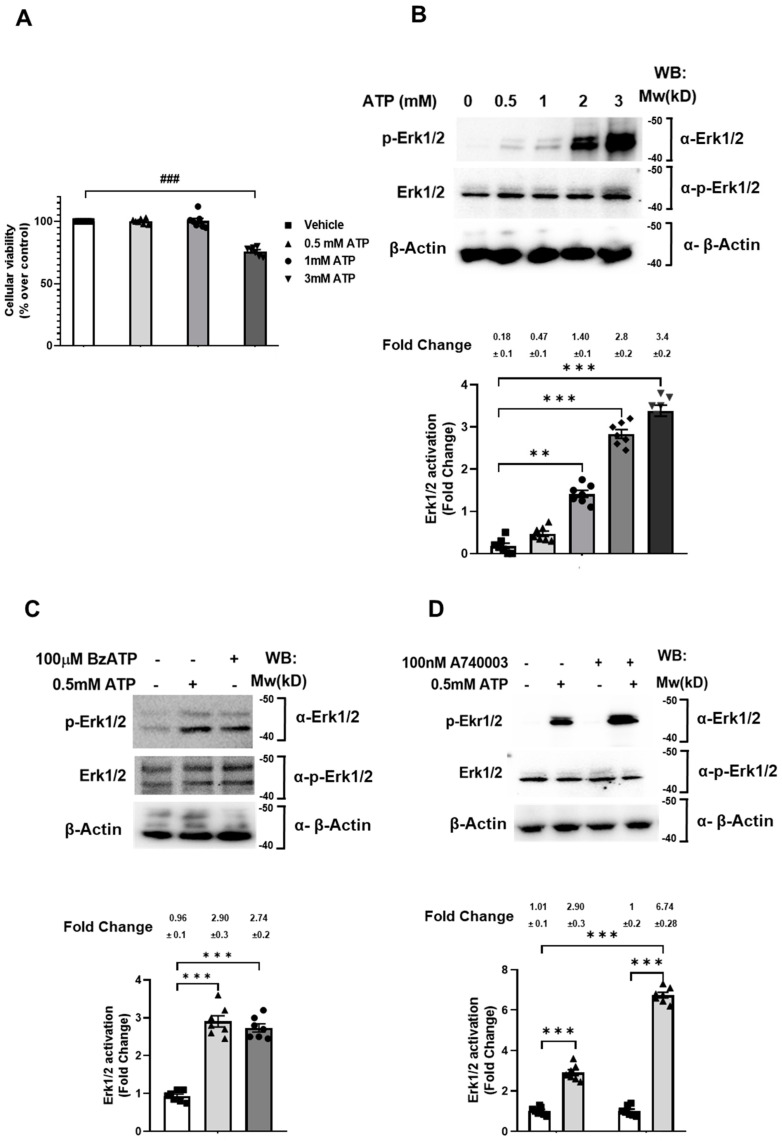
The ATP-stimulated P2X_7_ receptor controls cellular viability and Erk1/2 phosphorylation in SN4741 cells. (**A**) SN4741 cells were serum-starved overnight and stimulated, or not, with different concentration of ATP for 5 min, and the cellular viability was measured by MTT assay. The values are represented as the percentage on control (untreated cells) and show the mean of five independent experiments ± SEM. SN4741 overnight-serum-starved cells were (**B**) stimulated, or not, with different concentrations of ATP for 5 min, (**C**) 0.5 mM ATP for 5 min or 100 µM BzATP for 10 min, (**D**) pre-treated with or without 100 nM A740003 (Table 1) for 1 h and, subsequently, stimulated, or not, with 0.5 mM ATP for 5 min, and lysed. Cell extracts were analyzed by Western blot using an anti-phospho-Erk1/2 antibody. The Erk1/2 amounts present in the samples analyzed were examined with an anti-Erk ½ antibody. The β-Actin amounts present in the samples analyzed in lower panel were examined with an anti-β-Actin antibody. Results show the mean of seven independent experiments ± SEM. Statistical analysis shows a significant difference (** *p* < 0.01, *** *p* < 0.001 and ### *p* < 0.001).

The P2X_7_ receptor activation has been widely associated to the cell death program in different cell types [26,27,28,29]. In Jun et al., it was reported that the P2X_7_ receptor stimulated with 3 mM ATP mediated the SN4741 cells necrotic death [30]. To examine whether SN4741 cellular viability depends on the ATP concentration, SN4741 cells were treated, or not, with different concentrations of ATP for 5 min, and an MTT assay was used to measure the cell’s viability. As shown in Figure 1A, SN4741 cellular viability was not affected by either 0.5 or 1 mM ATP. However, 3 mM ATP significantly reduced cellular viability (Figure 1A). This result allowed us to establish 0.5 mM ATP as the working concentration to investigate the P2X_7_ receptor intracellular signaling.

Next, we investigated the relationship between purinergic receptors and Erk activation, which seems to play an important role in the regulation of both physiological and pathological cellular responses [31]. Thus, SN4741 cells were treated with different concentrations of ATP for 5 min. This was followed by SDS-PAGE separation of protein lysates and Western blot analysis of Erk1/2 phosphorylation. ATP of 0.5 mM is the minimum concentration capable of inducing maximum Erk1/2 phosphorylation (Figure 1B (second lane (0.47) compared with first lane (0.18)). It must also be taken into account that this concentration of ATP is not toxic for SN4741 cells, as shown in Figure 1A.

To demonstrate that P2X_7_ receptor was involved in Erk1/2 phosphorylation in SN4741 cells, the most specific P2X_7_ receptor agonist, BzATP (2,3-O-(4-benzoyl benzoyl)-ATP), was used [32]. Thus, SN4741 cells were treated, or not, with 0.5 mM ATP or with 100 µM BzATP for 5 min and lysed. This was followed by SDS-PAGE separation of protein lysates and Western blot analysis of Erk1/2 phosphorylation. The results show that BzATP stimulated Erk1/2 phosphorylation equally well as ATP (Figure 1C, upper panel, lanes 2 (2.9) and 3 (2.7) and histogram bars 2 and 3 (2.9 and 2.7, respectively)). In addition, we used A740003, a highly selective P2X_7_ receptor antagonist compared to other P2 receptors [33]. SN4741 cells were pre-treated, or not, with 100 nM A-740003 (Table 1) for 1 h and treated, or not, with 0.5 mM ATP for 5 min and lysed. This was followed by SDS-PAGE separation of protein lysates and Western blot analysis of Erk1/2 phosphorylation. As shown in Figure 1D, the antagonist not only did not block but enhanced Erk phosphorylation (upper panel lane and bar 4 (6.74) compared to lane and histogram bar 2 (2.9)). These results suggest that P2X_7_ receptor activation does not mediate ATP-stimulated Erk phosphorylation.

### 2.2. P2X_7_ Receptor Specific Control of Ras Activation in SN4741 Cells

**Figure 2:**
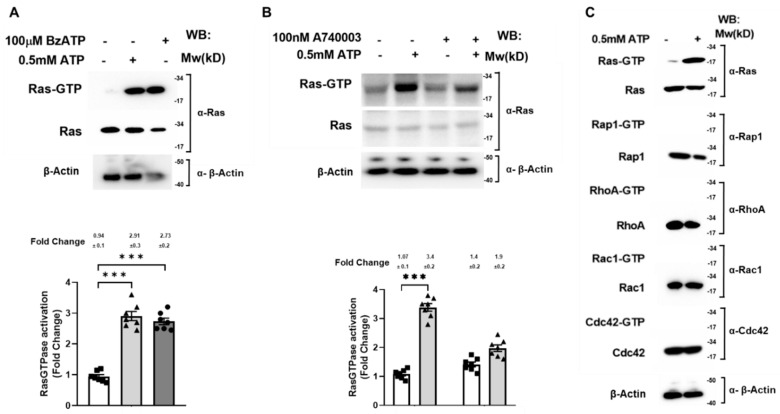
The P2X_7_ receptor-mediated specific Ras activation in SN4741 cells. The SN4741 cells were serum-starved overnight and treated, or not, with 0.5 mM ATP for 5 min and lysed. (**A**) Cell lysates were used to measure Ras, Rap1, RhoA, Rac1, and Cdc42 activation by the affinity precipitation assay. The precipitated active GTPases (binds to GTP) and the total GTPases expression levels were analyzed by Western blotting using a specific antibody. The results are representative of five independent experiments. (**B**) Serum-starved SN4741 cells were treated, or not, with 0.5 mM ATP for 5 min, and 100 µM BzATP for 10 min, or (**C**) previously treated with or without 100 nM A740003 for 1 h before a 5-min stimulation, or not, with 0.5 mM ATP and lysed. Cell lysates were used to measure Ras activation by the affinity precipitation assay. The precipitated active Ras (Ras-GTP) and the total Ras expression level were analyzed by Western blotting using an anti-Ras antibody. The β-Actin amounts present in the samples analyzed in lower panel were examined with an anti-β-Actin antibody. Immunoblots are representative of seven independent experiments. Each histogram bar includes the values of five different samples. All values are expressed as fold change. The differences between treatments were *p* < 0.001 in all cases (***).

The Ras GTPase-mediated canonical activation of Erk prompted us to find out what is the state of Ras in conditions similar to those described for Erk1/2. Thereby, SN4741 cells were treated, or not treated, with 0.5 mM ATP or with 100 µM BzATP for 5 min. Cells were lysed, and the endogenous active Ras was measured by the pulldown assay, as described in Materials and Methods section. As shown in Figure 2A, ATP stimulated the activation of Ras GTPase as strongly as BzATP (Table 1), the P2X_7_ receptor most specific agonist (upper panel and histogram lane 2 (2.91) and 3 (2.73)). To verify that the P2X_7_ receptor was specifically involved in Ras activation, SN4741 cells were previously treated, or not, with 100 µM A-740003 (Table 1) for 1 h and treated, or not, with 0.5 mM ATP for 5 min. SN4741 cells were lysed, and the endogenous active Ras was measured by the pulldown assay. Results show that ATP stimulated Ras activation, as expected, but A-740003, a P2X_7_ receptor antagonist, strongly reduced the ATP-stimulated Ras activation (Figure 2B (upper panel lane and bar 4 (1.9) compared to lane and histogram bar 2 (3.4)). This finding suggests that, unlike Erk activation, P2X_7_ receptor could mediate Ras activation, at least partially.

Going further, we examined whether other GTPases of the Ras family, such as Rap1, or of the Rho family, such as RhoA, Rac1, and Cdc42, could be activated under the same conditions as Ras in SN4741 cells. To this end, SN4741 cells were treated, or not, with 0.5 mM ATP for 5 min, then cells were lysed, and the endogenous active Rap1, RhoA, Rac1, and Cdc42 were captured with the pull-down assay and measured, as described in the Materials and Methods section. Except for Ras, none of the other GTPases was activated after ATP stimulation of SN4741 cells (Figure 2C).

### 2.3. P2X_7_ Receptor-Specific Control of Ras Activation Is via the Ca^2+^-Calmodulin/RasGRF1 Signaling Pathway

Next, we aimed to identify the guanine nucleotide exchange factor (GEF) that preferentially activates Ras in our cellular system. Assuming that RasGRF1 is one of the GEFs that mediates Ras activation, and it is highly expressed in the central nervous system [34], we selected the easily transfected HEK 293T cell system to investigate P2X_7_ receptor and RasGRF1 functions since these cells do not express either the P2X_7_ receptor or RasGRF1 [35,36].

**Figure 3:**
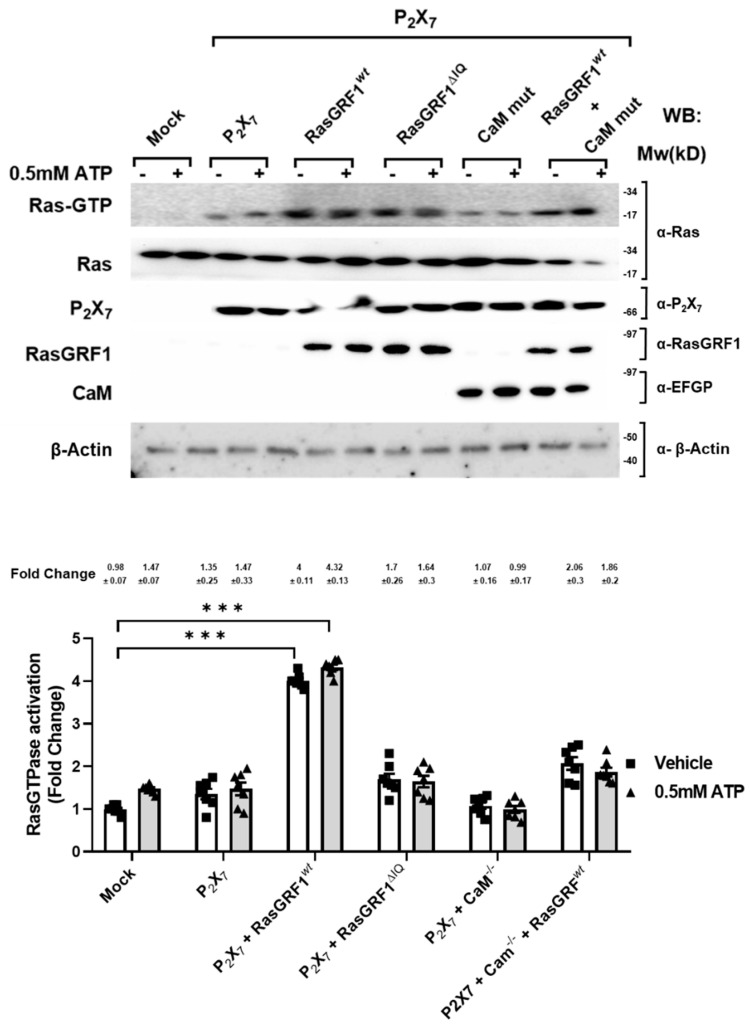
The P2X_7_ receptor requires CaM/RasGRF1 pathway to activate Ras in HEK 293 T cells. The HEK 293 T cells were transfected with empty vector (mock control), pCELF-HA-P2X_7_ receptor, or co-transfected with the P2X_7_ receptor, together with pEGFP-RasGRF1wt, pEGFP-RasGRF1mutIQ, or pcDNA3YFP-CaM mut, or with a combination of the pEGFP-RasGRF1wt and pcDNA3YFP-CaM mut. Forty-eight hours post-transfection, cells were treated, or not, with 0.5 mM ATP for 5 min and lysed. Cell lysates were used to measure Ras activation by the affinity precipitation assay. The precipitated active Ras (Ras-GTP), total Ras, P2X_7_ receptor, RasGRF1wt, RasGRF1mutIQ, and CaM mut expression levels were analyzed by Western blotting using specific antibodies. The β-Actin amounts present in the samples analyzed in lower panel were examined with an anti-β-Actin antibody. Immunoblots are representative of five independent experiments. Each histogram bar includes the values of seven different samples. All values are expressed in fold change. The differences between treatments were *p* < 0.001 in all cases (***).

HEK 293T cells were co-transfected with the pCEFL-HA-P2X_7_ receptor and pEGFP-RasGRF1(wt) or pEGFP-RasGRF1^R214Q, R219Q, K223Q^ (pEGFP-RasGRF1 mut-IQ), or pCEFL-HA-P2X_7_ with pcDNA3YFP-CaM mut or pCEFL-HA-P2X_7_ receptor with pcDNA3CaM mut and pEGFP-RasGRF1 (wt), as indicated in Figure 3. After 48 h, cells were treated, or not, with 0.5 mM ATP for 5 min and lysed, and endogenous active Ras was captured with the pull-down assay and measured, as described in the Materials and Methods. As expected, ATP did not induce Ras activation either in non- or P2X_7_ receptor transfected cells (Figure 3, first panel, lanes 2 and 4, and histogram bars 2 and 4 (both 1.47)); however, the P2X_7_ receptor/ RasGRF1(wt) co-expression rendered possible a robust increase in Ras activation in both ATP-treated and untreated cells (Figure 3, first panel, lanes and bars 5 (4) and 6 (4.32) compared to lanes and bars 1, 2, 3, and 4 (0.98–1.47)). We hypothesize that the Ras activation in untreated cells was due to the overexpression of RasGRF1. In general, it is well accepted that GEF overexpression per se is enough to mediate the small GTPase activation. Furthermore, the IQ domain RasGRF1 mutant (RasGRF1 mut-IQ) responsible for the binding to Calmodulin was unable to mediate Ras activation, even in the presence of P2X_7_ receptor (Figure 3, first panel, lanes and bars 7 (1.7) and 8 (1.64) compared to lanes and bars 5 (4) and 6 (4.32)). This finding suggested that the calmodulin-binding domain of RasGRF1 was necessary for the correct signal transduction. To verify this result, the P2X_7_ receptor and RasGRF1(wt) were co-transfected, together with a calmodulin mutant form (CaM mut), unable to bind calcium. As shown in Figure 3, CaM mut efficiently blocked Ras activation both in treated and untreated cells (lanes and bars 11 (2.06) and 12 (1.86) compare to lanes and bars 5 (4) and 6 (4.32)). Altogether, these results suggest that the P2X_7_ receptor recruits in tandem Ca^2+^-Calmodulin/RasGRF1 to activate Ras. 

### 2.4. Tyrosine Phosphatases Regulate Ras/Erk Activation and Control SN4741 Cell Viability via the P2X_7_ Receptor

**Figure 4:**
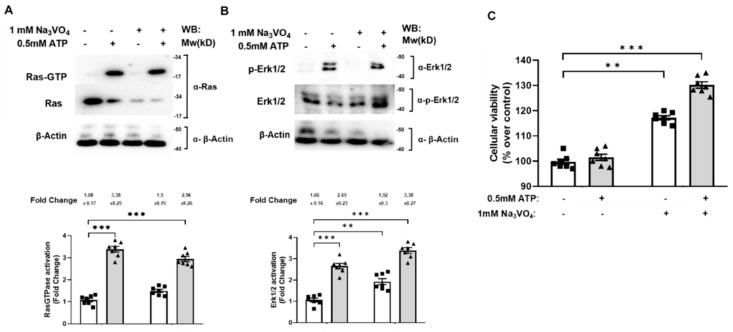
The tyrosine phosphatases mediate Ras/Erk1/2 activation and control SN4741 cells viability. SN4741 cells were serum-starved overnight, and cells were previously treated, or not, with 1 mM Na_3_VO_4_ for 20 min and subsequently stimulated, or not, with 0.5 mM ATP for 5 min and lysed. Cell lysates were used to measure (**A**) Ras activation by the affinity precipitation assay and (**B**) ERK1/2 phosphorylation in total extracts. The precipitated active Ras (Ras-GTP), p44/42 phosphorylation, total Ras, Erk ½, and β-Actin expression levels were analyzed by Western blotting using specific antibodies. Immunoblots are representative of four independent experiments. Each histogram bar includes the values of four different samples. All values are expressed in fold change. The differences between treatments were *p* < 0.001 in all cases (***) (Mann–Whitney test). (**C**) SN4741 cells were serum-starved overnight and were treated, or not, with 1 mM Na_3_VO_4_ for 20 min and subsequently stimulated, or not, with 0.5 mM ATP for 5 min, and cellular viability was measured by the MTT assay. All values are represented as the percentage over control (untreated) cells and show the mean of seven independent experiments ± SEM. Statistical analysis shows a significant difference (** *p* < 0.01 and *** *p* < 0.001).

The reported basal phosphatase-activity mediating the P2X_7_ receptor dephosphorylation [16] prompted us to investigate whether tyrosine phosphatase activity is involved in controlling the P2X_7_ receptor signaling in SN4741 cells. For this purpose, SN4741 cells were previously treated, or not, with 1 mM of the tyrosine phosphatase inhibitor sodium orthovanadate (Na_3_VO_4_) (Table 1) for 20 min and treated, or not, with 0.5 mM ATP for 5 min and lysed. Endogenous active Ras was captured with the pull-down assay and measured, as described in the Materials and Methods. As expected, ATP induced strong Ras activation, while Na_3_VO_4_ did not affect it (Figure 4A upper panel lanes 2 (3.38), 4 (2.94) and bars 2, 4)). Regarding Erk1/2, cell protein lysates were separated by SDS-PAGE, and Erk1/2 phosphorylation was analyzed by Western blot. As shown in Figure 4B, ATP induced robust Erk1/2 phosphorylation, and Na_3_VO_4_ did not affect it (upper panel lanes 2, 4 and bars 2, 4 (2.65 and 3.38, respectively)). However, in the absence of ATP stimulus, Na_3_VO_4_ significantly enhanced Erk1/2 phosphorylation (Figure 4B, upper panel lane 3, bar 3 (1.92)). 

Furthermore, we examined the tyrosine phosphatases potential role in SN4741 cell viability. Cells were previously treated, or not, with 1 mM Na_3_VO_4_ for 20 min and treated, or not, with 0.5 mM ATP for 5 min, and the MTT assay measured cell viability. As shown in Figure 4C, ATP did not alter the cell viability, while Na_3_VO_4_ strongly increased cell viability both in the absence and in the presence of ATP, i.e., between 20 to 30% higher than control (Figure 4C, bars 3 and 4). All these findings suggest that tyrosine phosphatases regulate RasGTPase/Erk activation and control cell viability in the P2X_7_ receptor-dependent manner.

### 2.5. PTPRβ Regulates Ras/Erk Activation and Controls SN4741 Cell Viability via the P2X_7_ Receptor

**Figure 5:**
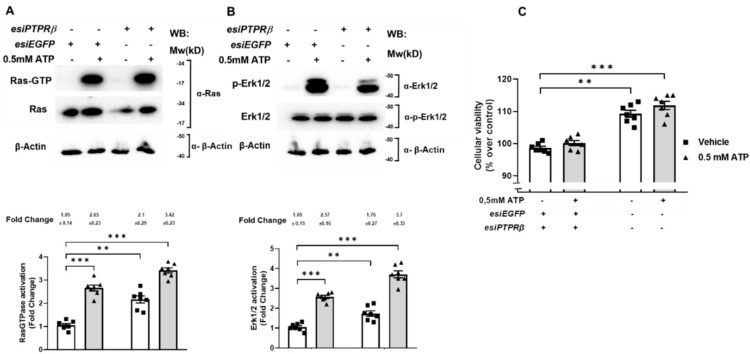
PTPRβ controls cellular viability and Ras and Erk1/2 activation in SN4741 cells. SN4741 cells were transfected with *esiPTPRβ* or esi*EGFP* as control. Twenty-four hours post-transfection, SN4741 cells were serum-starved overnight. Then, cells were treated, or not, with 0.5 mM ATP for 5 min and lysed. The cell lysates were used to measure Ras activation by the affinity precipitation assay and Erk1/2 phosphorylation in total extracts. (**A**) The precipitated active Ras (Ras-GTP), (**B**) p44/42 phosphorylation, and the total Ras, Erk1/2 and β-Actin expression levels were analyzed by Western blotting using specific antibodies. Immunoblots are representative of four independent experiments. Each histogram bar includes values of three different samples. All values are expressed in fold change. Differences between treatments were *p* < 0.01 (**) or *p* < 0.001 (***) (Mann–Whitney test). (**C**) SN4741 cells were transfected with *esiPTPRβ* or esi*EGFP* as control. Twenty-four hours post-transfection, SN4741 cells were serum-starved overnight and stimulated, or not, with different 0.5 mM ATP for 5 min, and cellular viability was measured by the MTT assay. Values are presented as the percentage of control (untreated) cells, and they are representative of five experiments. Cells were serum-starved (1% FBS) overnight and previously treated, or not, with 1 mM Na_3_VO_4_ for 20 min and subsequently stimulated, or not, with 0.5 mM ATP for 5 min, and cellular viability was measured by the MTT assay. Values are presented as the percentage over control (untreated) cells and represent the mean of five independent experiments ± SEM. Statistical analysis shows a significant difference (** *p* < 0.01 and *** *p* < 0.001).

Our results, together with the well-described molecular binding between the P2X_7_ receptor and the tyrosine phosphatase beta (PTPRβ) [16], prompted us to examine a putative role of PTPRβ in the P2X_7_ receptor signaling leading to Ras/Erk activation and SN4741 cell viability control. We approached the question genetically by knocking down PTPRβ in SN4741 cells. To this end, SN4741 cells were transfected with esiRNA mouse PTPRβ or esiRNA targeting EGFP as a negative control. Twenty-four hours post-transfection, Ras activation was determined in both SN4741 cells stimulated with ATP for 5 min and unstimulated. As shown in Figure 5A, ATP stimulated robust Ras activation in egfp(esiRNA)-transfected SN4741 cells. Non-stimulated ptprβ-knockdown (ptprβ(esiRNA)) cells exhibited increased Ras active configuration (upper panel, lane and bar 4 (3.42) compared to lane and bar 2 (2.65)). When we measured the Erk phosphorylation state, under the same conditions as Ras, we obtained the same results; the absence of ptprβ expression enhanced Erk phosphorylation (Figure 5B, upper panel lane and bar 4 (3.7) compared to lane and bar 2 (2.57)). 

Last, we investigated whether the absence of PTPRβ expression modified SN4741 cells viability. SN4741 cells were transfected with esiRNA mouse PTPRβ or esiRNA targeting EGFP as a negative control. Twenty-four hours post-transfection, cell viability was determined in both SN4741 cells stimulated with ATP for 5 min and unstimulated by MTT assay. As shown in Figure 5C, ATP did not modify cell viability (compare bar 2 to bar 1). However, hptprβ-knockdown SN4741 cells displayed enhanced viability, even in the absence of a stimulus (Figure 5C, bars 3 and 4). Taken together, those findings suggest that tyrosine phosphatase PTPRβ mediate RasGTPase/Erk activation and control cell viability in a P2X_7_ receptor-dependent manner.

## 3. Discussion

Our study reveals the purinergic P2X_7_ receptor modulates positively neuronal cell survival via Calcium-Calmodulin pathway regulation of the RasGRF1-mediated Ras GTPase/Erk1/2 activation and negatively via the tyrosine phosphatase, hPTPβ.

Purinergic signaling has been pointed out as the mediating mechanism for dopaminergic neuronal death [30] and may constitute an initial event in the development of Parkinson’s disease (PD). Several other studies have reported that the viability of the SN4741 cell line decreased dramatically after stimulation of purinergic receptors with 3 mM ATP for 20 min [30,37,38,39]. However, our results show that reducing the ATP concentration to 0.5 mM, six times lower than reported, not only preserves cell viability, but this concentration also effectively activates Ras and Erk1/2. 

Considering that ATP and BZATP stimulation may activate several different purinergic receptors, such as P2X_1_, P2X_3_, P2X_4_, P2X_7_, and P2X_2_, and our pharmacological approach, using the P2X_7_ receptor-specific antagonist (A740003), ruled out this receptor as the sole activator of Erk1/2. Although it is not the only receptor, it is one of the major mediators of Ras activation. According to the results obtained, we postulated that ATP binds to the P2X_7_ and other P2X receptors. So, ATP binding to the P2X_7_ receptor mainly activates the Ras GTPase. Likewise, Ras activation is not affected by the presence of a specific antagonist (A740003) interfering with the binding capacity of the ligand to the P2X_7_ receptor. In contrast, Erk1/2 activation is partially affected, which is consistent with the fact that Erk1/2 can also be activated by Rap1/Raf pathway [40], Rho/Rho kinase (Rock) [41], and PKCs [42], different subtypes of G-protein alpha subunits, such as Gα12, Gαs, Gαq, and Gαi/o, or by beta-arrestin [43], in a Ras GTPases independent manner.

Ionotropic purinergic receptors, including the P2X_7_ receptor, modulate calcium fluxes that are key in neuronal physiology. Regarding Ras GTPase activation in neurons, there are two families of Ras GEFs that are associated with calcium fluxes: RasGRP and RasGRF. RasGRP has two EF domains that could bind calcium; however, the participation of Ca^2+^ as a RasGRP regulator has not been clearly demonstrated. The EF domains of this GEF do not seem to act as a typical calcium sensor. It is thought that they could play a role in the association of proteins to the plasma membrane [44]. Although Ras GRF1 activity does not depend on calcium movements directly, it depends on calmodulin. This has been demonstrated in a very elegant paper by Farnsworth et al. using Hek cells that do not express Ras GRF1 [36]. Our results are in complete agreement with those of Farnsworth et al., in which they also pointed out the calcium-calmodulin-mediated Ras activation via RasGRF1. 

The Ras/MAPK signaling cascade may be considered a pathway that favors cell survival [45], although it has also been suggested that it can promote cell death [31,46,47]. Several models of neuronal toxicity associated with Parkinson’s disease show greater Erk1/2 activity present mainly in the cytoplasm and mitochondria accompanied by an increase in mitophagy [48,49,50]. While low levels of mitophagy confer neuroprotection, excessive activity due to increased phosphorylation of ERK is detrimental to the functionality of neurons.

The P2X_7_ purinergic receptor interacts with at least 11 proteins, including tyrosine phosphatase PTPRβ, a phosphatase whose expression is restricted to the central nervous system. The regulatory activity of this phosphatase on the anaplastic lymphoma protein kinase ALK has also been shown [51,52,53,54]. It should be noted that ALK activation has been associated with neuronal differentiation and neurite growth mediated by the MAPK pathway [55,56,57,58]. We observed that tyrosine phosphatases participate in the circuitry controlling Ras activation in SN4741 cells. This tyrosine phosphatase control of Ras activation coincides with that described by Kim et al., in which they reported that a basal phosphatase activity dephosphorylated the P2X_7_ receptor [16]. Our results suggest the participation of different phosphatases, where PTPRβ could display basal activity in neurons, while it would be a still unidentified phosphatase that increases its activity after purinergic stimulation. In this way, this increased phosphatase activity could act via the purinergic receptors themselves, as previously described [16], or via a receptor-independent mechanism, in any case, promoting Ras activation.

However, the regulation of Ras and Erk1/2 activity is not the only role of tyrosine phosphatases in dopaminergic neurons. These phosphatases activities promote other effects aiming at neuronal death. Our results show that the pharmacological inhibition of tyrosine phosphatases prevents ATP-stimulated death of dopaminergic neurons. This neuronal death protective effect via the inhibition of tyrosine phosphatases has already been described in the 6-OHDA-induced PD model [59], which pointed out these phosphatases as potential therapeutic targets. We focused on the role of PTPRβ tyrosine phosphatase, which, without being the only one involved in neuronal cell death when knocked-down, a blockage of SN4741 cell death was observed again.

**Figure 6:**
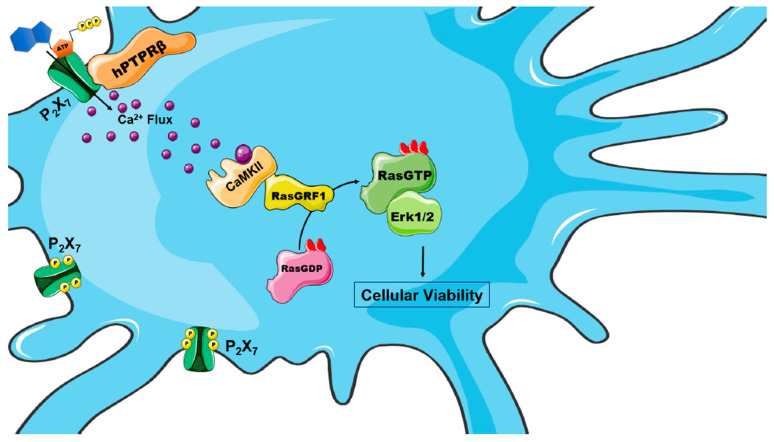
Low ATP concentrations promote cell survival via the CamKII/RasGRF1/RasGTPase/Erk pathway via the P2X_7_/hPTPB receptor system in SN4741 dopaminergic neurons. Scheme of the signaling cascade promoted by ATP stimulation of CamKII/RasGRF1/RasGTPase/Erk via the P2X_7_ receptor that promotes cell survival in SN4741 dopaminergic neurons. Low concentrations of extracellular ATP activate the P2X_7_ receptor. In a basal state of activation, the P2X_7_ receptor is phosphorylated. Due to the action of hPTPB tyrosine phosphatase, the P2X_7_ receptor is dephosphorylated, causing a greater flow of calcium, that promotes the activation of CamKII. Active CamKII binds to IQ domain of the RasGRF1 nucleotide exchanger. RasGRF1 promotes the change of RasGTPase from the inactive state (GDP-bound Ras) to the active state (GTP-bound Ras). Activated Ras can activate its main effector molecule Erk1/2. According to our results presented here, this described signaling cascade is involved in the cell survival of SN4741 dopaminergic neurons.

In conclusion, as shown in Figure 6, our findings reveal the mechanism through which P2X_7_ receptor stimulates Ras/Erk1/2 activation in SN4741 cells. Besides, we identified RasGRF1 as the GEF that specifically activates Ras, as well as the molecular mechanism of RasGRF1 activation of the P2X_7_/Ras pathway. Regulation of this novel signaling cascade requires the hPTPβ tyrosine phosphatase participation in the SN4741 dopaminergic neuronal cell viability. Therefore, we postulate that neuronal cell survival could be promoted by targeting hPTPβ. Future studies on this cellular context will help us clarify these cellular survival mechanisms and also find new therapeutic targets to prevent the death of dopaminergic neurons in Parkinson’s disease.

## 4. Experimental Procedures

### 4.1. Reagents

The following reagents were from Sigma-Aldrich (Lyon, France): DMSO, BzATP (2′(3′)-O-(4-Benzoylbenzoyl) adenosine 5′-triphosphate triethylammonium salt), A-740003 (N-(1-{[(cyanoimino) (5-quinolinylamino)methyl]amino}-2,2-dimethylpropyl)-2-(3,4dimethoxy phenyl)acetamide), GF109203X hydrochloride (3-[1-(Dimethylaminopropyl)indol-3-yl]-4-(indol-3-yl)maleimidehydrochloride, Bisindolylmaleimide I hydrochloride), sodium orthovanadate (Na_3_VO_4_), MISSION^®^ esiRNA targeting EGFP (reference EHUEGFP) (EGFP was used as a negative transfection control because it was absent in our cellular system), and MISSION^®^ esiRNA targeting human hPTPRβ (reference EHU158501).

Mouse monoclonal anti-RAS clone RAS10 antibody (Dil. 1:1000), anti-Cdc42 antibody (Dil. 1:1000), and anti-Rac clone23A8 (Dil. 1:1000) antibody were obtained from Millipore (Burlington, MA, USA). Anti-RAP1 sc:65 antibody (Dil. 1:1000) was obtained from Santa Cruz Biotechnology, Inc. (Dallas, TX, USA); rabbit monoclonal anti-Erk1/2 (Dil. 1:1000) and anti-phospho-Erk1/2 (Dil. 1:1000) were obtained from Cell Signaling Technology (Danvers, MA, USA). Monoclonal Anti-β-Actin antibody produced in mouse (Dil. 1:5000) were obtained from Cell Signaling Technology (Massachusetts, USA). The Clarity enhanced chemiluminescence (ECL) reagent was obtained from BioRad Laboratories Inc (Madrid, Spain).

### 4.2. Cell Culture and Transfections

SN4741 cells were cultured in DMEM (Dulbecco’s Modified Eagle Medium, Sigma-Aldrich), supplemented with 10% fetal bovine serum (FBS) (Sigma-Aldrich), 2 mM L-glutamine (Sigma-Aldrich), 100 units/mL of penicillin, and 100 ng/mL of streptomycin (Sigma-Aldrich) at 33 °C in a 5% CO_2_ atmosphere and a 95% relative humidity, as indicated by Son et al. [25]. For transfections, SN4741 cells were harvested at a rate of 8 × 10^6^ cells/200 µL of complete medium previously chilled on ice. For each experimental point, 15 ng of esiRNA (MISSION^®^ esiRNA, Sigma-Aldrich) were added to a previously cooled electroporation cuvette and then 200 µL of the cell suspension were added. Electroporation was performed in a Gene Pulser Xcell Electroporator (Bio-Rad) at 260 V and 950 µF [60]. Subsequently, transfected cells were brought into 10 mL of complete growth medium equilibrated at 33 °C in a 100 mm diameter plate. After 24 h of incubation, the medium was changed, and cells were treated to continue the experiment.

Human embryonic kidney (HEK) 293 cells were maintained in Dulbecco’s modified Eagle’s medium supplemented with 10% fetal bovine serum (FBS), 1% Penicilin/Streptomicin, and 1% Sodium pyruvate at 37 °C in a 5% CO_2_ atmosphere and a 95% relative humidity. A low passage number (0 to 20) of cell lines were maintained in 100-mm plates for adherent cells (Sarstedt, Nümbrecht, Germany). For plasmid and esiRNA transfections, HEK293 cells were seeded at 300.000 cells/well. The transfection was performed using XtremeGENE HD Roche (Paris, France) transfection reagent following manufacturer′s recommendations. To silence endogenous expression of human hPTPRβ to inhibit endogenous microRNA activity, cells were transfected with commercial esiRNAs following manufacturer’s recommendations. MISSION^®^ esiRNA targeting EGFP (reference EHUEGFP) (EGFP was used as a negative transfection control because it was lacking in our cellular system) was used, as well as MISSION^®^ esiRNA targeting human hPTPRβ (reference EHU158501)

### 4.3. Ras, Rap1, Rho, Rac1, and Cdc42 Activation Assay

Ras and Rap1 pull-down assay was performed using a GST fusion protein containing the RAP1-binding domain of Ral guanine nucleotide dissociation stimulator (GST-RalGDS) [61]. Rac1 pull-down assay was performed using a GST fusion protein containing the Rac1 binding domain of PAK1 (GST-RBD-PAK1) [62]. Cdc42 pull-down assay was performed using a GST fusion protein containing the Cdc42/Rac1 interactive binding domain of WASP (GST-CRIB-WASP) [63].

Cells were transfected with the indicated constructs or previously treated with specific inhibitors and, finally, lysed in 500 μL of lysis buffer (10 mM Tris, pH 7.6, 150 mM NaCl, 1% IGEPAL-360, 10 mM MgCl_2_, 1 mM PMSF, 10 μg/mL aprotinin, 10 μg/mL leupeptin) [62]. Cell lysates were centrifuged at 13,500 rpm for 10 min at 4 °C, and 500 μg of protein (1 μg/μL) from the soluble fraction was incubated for 1 h at 4 °C with 50 μg of the GST-RBD-specific fusion protein. Precipitated proteins were eluted from beads using 2× loading buffer (12 mm Tris, pH 6.8, 5% glycerol, 0.4% SDS, 140 mm 2-mercaptoethanol, and 0.02% bromophenol blue), separated by SDS-PAGE, and analyzed by Western blotting with specific monoclonal antibodies. Immunoreactive bands were visualized using the ECL reagent. Band intensities were imaged using ChemiDoc MP Imaging System, and the images were analyzed with Image Lab 5.0. Software (Bio-Rad).

### 4.4. Cell Viability Assay

SN4741 cells were seeded in triplicate at 30 × 10^3^ cells/well in a 96-well plate. Then, cells were previously treated (+), or not (−), with A-740003, GF109203X hydrochloride, or sodium orthovanadate. The next day, cells were treated, which was stopped by removing the medium and washing cells with cold 1X PBS. One percent FBS medium was added to avoid cellular proliferation during the test. Cell viability was measured with CellTiter 96 Non-Radioactive Cell Proliferation Assay (MTT) following the manufacturer’s instructions (Promega, Madison, WI, USA): Briefly, 10 µL MTT reagent (MTT labeling reagent) was added to the fresh medium in each well. The plate was incubated at 33 °C in 5% CO_2_ for 4 h, and the reaction was stopped by adding 100 µL of kit solubilization solution to each well overnight at 33 °C, and 5% CO^2^. The absorbance was measured in a microplate reader (Synergy™ HT Multidetection Microplate Reader, BIOTEK (Winooski, VT, USA)) at the wavelength of 570 nm. The values obtained were transformed into percentages, setting the value of 100% viability to the net absorbance of control cells. The rest of the values corresponding to different treatments are presented as percentages over the control.

To verify the MTT assay, we counted with a Neubauer counting chamber with cover glasses (dimensions are 24 mm L × 26 mm W × 0.4 mm H) (Blau brand). Cells were counted under the microscope. Sample preparation was done by an experienced senior laboratory personnel researcher. In order to reduce the errors caused by subjective central tendency. The samples were diluted with Trypan blue solution (Sigma-Aldrich) in a normal phosphate buffer saline at 1:5 ratio.

### 4.5. Statistical Analysis

All data are presented as mean ± S.E.M. Statistical analysis was performed using GraphPad Prism statistical software (version 5.0; GraphPad software (San Diego, CA, USA)). Comparisons between multiple experimental groups were made using one-way analysis of variance (ANOVA), followed by Bonferroni’s post hoc test. To determine the significance between data means, (* *p* < 0.05, ** *p* < 0.01, *** *p* < 0.001).

## Figures and Tables

**Table 1:** List of agonists and inhibitors and concentration used.

Drug Name	Activity	Treatment	Concentration
ATP	Agonist of P2X receptors	5 min	500 µM
BzATP	Specific agonist of P2X_7_ receptor	10 min	100 µM
A-740003	Specific antagonist of P2X_7_ receptor	30 min	100 nM
GF109203X hydrochloride	PKC inhibitor	1 h	1 µM
Na_3_VO_4_	Tyrosine phosphatase inhibitor	20 min	1 mM

## Data Availability

The datasets generated during and analyzed during the current study are available from the corresponding author or principal investigator (last author) on reasonable request.

## Abbreviations

ATP—Adenosine triphosphate; CaM—Ca2+/calmodulin (CaM)-dependent protein kinase II; CNS—Central nervous system; DA—Dopaminergic; DMSO—Dimethyl sulfoxide; Erk1/2—extracellular signal-regulated kinase ½; GAP—GTPase-activating proteins; GDI -Guanosine nucleotide dissociation inhibitor; GDP—guanosine diphosphate; GEF—Guanine nucleotide exchange factors; GST—Glutathione S transferase; GTP—guanosine triphosphate; JNKs—Jun N-Terminal Kinase; MAPKs—Mitogen Activated Protein Kinases; Na3VO4—Sodium Orthovanadate; PAK1—PAK1 Serine/threonine-protein kinase 1; PD—Parkinson’s disease; Raf—Raf Serine/threonine-protein kinase; RasGRF1—Ras Protein Specific Guanine Nucleotide Releasing Factor 1; ROS—reactive oxygen species; PTPRβ—Protein Tyrosine Phosphatase Receptor β; SDS—Sodium dodecyl sulfate; SDS-PAGE—sodium dodecyl sulfate polyacrylamide gel electrophoresis; WASP—Wiskott–Aldrich Syndrome protein.
